# Microrheological comparison of melanoma cells by atomic force microscopy

**DOI:** 10.1007/s10867-023-09648-w

**Published:** 2024-01-19

**Authors:** M. Manuela Brás, Aureliana Sousa, Tânia B. Cruz, Jonas Michalewski, Marina Leite, Susana R. Sousa, Pedro L. Granja, Manfred Radmacher

**Affiliations:** 1 Instituto de Investigação e Inovação em Saúde (i3S), Universidade do Porto, Porto, 4200-135 Portugal; 2 Faculdade de Engenharia da Universidade do Porto (FEUP), Porto, 4200-465 Portugal; 3 Institute of Biophysics, University of Bremen, Bremen, 28334 Germany; 4 Instituto Superior de Engenharia do Porto (ISEP), Instituto Politécnico do Porto, Porto, 4200-072 Portugal

**Keywords:** Melanocytes, Melanoma, Microrheology atomic force microscopy, AFM, Viscoelasticity, Frequency sweep, Power-law exponent

## Abstract

Melanoma is one of the most severe cancers due to its great potential to form metastasis. Recent studies showed the importance of mechanical property assessment in metastasis formation which depends on the cytoskeleton dynamics and cell migration. Although cells are considered purely elastic, they are viscoelastic entities. Microrheology atomic force microscopy (AFM) enables the assessment of elasticity and viscous properties, which are relevant to cell behavior regulation. The current work compares the mechanical properties of human neonatal primary melanocytes (HNPMs) with two melanoma cell lines (WM793B and 1205LU cells), using microrheology AFM. Immunocytochemistry of F-actin filaments and phosphorylated focal adhesion kinase (p-FAK) and cell migration assays were performed to understand the differences found in microrheology AFM regarding the tumor cell lines tested. AFM revealed that HNPMs and tumor cell lines had distinct mechanical properties. HNPMs were softer, less viscous, presenting a higher power-law than melanoma cells. Immunostaining showed that metastatic 1205LU cells expressed more p-FAK than WM793B cells. Melanoma cell migration assays showed that WM73B did not close the gap, in contrast to 1205LU cells, which closed the gap at the end of 23 h. These data seem to corroborate the high migratory behavior of 1205LU cells. Microrheology AFM applied to HNPMs and melanoma cells allowed the quantification of elasticity, viscous properties, glassy phase, and power-law properties, which have an impact in cell migration and metastasis formation. AFM study is important since it can be used as a biomarker of the different stages of the disease in melanoma.

**Supplementary Information:**

The online version contains supplementary material available at 10.1007/s10867-023-09648-w.

## Introduction

Melanoma is one of the most severe skin cancers in humans due to its high metastatic potential, with a mortality rate of 80% [1–4]. This type of cancer usually forms metastasis, via blood or the lymphatic system, to several organs such as the skin, lung, brain, liver, bone, and intestine [5]. Melanoma is originated from the malignant transformation of melanocytes, which are the normal pigment-producing cells located at the stratum basal of the epidermis [6]. Malignant melanocytes are characterized by changes in biophysical properties, like shape, elasticity, and stiffness, which contribute to cell motility, invasion, and metastasis formation [7]. In the latter case, the plasticity of melanocytes is very important since, for that purpose, they need to be highly deformable [8].

In order to form metastasis, tumor cells may detach from the primary tumor, invade blood or lymphatic vessels, extravasate from them, and establish new tumors at different sites of the primary one. To a large extent, the actin cytoskeleton is responsible for the elasticity of cells since it experiences assembly and disassembly during cell motion, regulating protrusion formation, focal adhesion behavior, and contractile filament organization. During migration of melanoma cells, integrins and focal adhesions play an important role since they establish the connection between the cell cytoskeleton with the extracellular matrix (ECM) [9, 10]. Focal adhesion kinase (FAK) plays a critical role in migration and invasion, involving integrin signaling and recruitment of the MT1 protease (a type of matrix metalloproteinases, MMP), which contributes to the ECM degradation required for tumor cell invasion [11–13]. A high amount of FAK expression in human cancer was correlated with high malignancy and invasiveness [11, 13].

The ECM, in particular its mechanics, is also known to contribute to cell migration since the ability of cells to migrate is influenced by stiffness gradients (from less stiff to stiffer substrates), a process called durotaxis [14, 15]. According to Huang et al*.*, for migration and metastasis, fluid shear stress (FSS) is also important and variable in the microenvironment of tumor metastasis-related fluid [16]. Accordingly, physical processes like FSS, friction, adhesion, and cell deformation contribute to the mobility of malignant melanocytes [16].

The assessment of the mechanical properties of cells has an increasingly prominent role in the understanding of various biological and biochemical mechanisms related to several cellular processes, such as migration and metastasis formation. This is particularly relevant for cancer cells that adapt their elasticity for an efficient migration [17]. In most published works, the quantification of cell mechanical properties is performed using static methodologies, recurring to Hertz’s model or its variants [18–20]. This model assumes that the cells are isotropic, homogenous, purely elastic, and a semi-infinite material, resulting in the determination of only an apparent Young’s modulus [21]. However, since cells are dynamic entities also exhibiting viscous properties, a more elaborated analysis is needed that includes the contribution of the viscoelastic nature of the cells, which have been neglected by the current methods of analysis.

In rheology, cells can be described as soft glassy materials, with properties varying according to time and frequency [22]. Some authors explain cell mechanical behavior through the application of a power-law exponent to the cells, which could be correlated with the “fluidity” of the cytosol [22–24].

The main goal of the present work was to quantify the elastic and viscous mechanical properties of cells and discuss their contribution to the migratory behavior of melanoma cells, which is relevant to metastasis. For that purpose, the same methodology used by Brás et al. consisting in the dynamic atomic force microscopy (AFM) frequency sweep was used, allowing the assessment of the storage and loss moduli, the power-law exponent applied to the storage modulus, and the loss tangent, which has not been performed in melanoma cells before [25]. The same methodology was applied to confirm that it can be used for different cancer cell types. Additionally, this technique allowed the detection of stress relaxation and creep of cells, as a function of time [26]. Mechanical properties of human neonatal primary melanocytes (HNPMs) and two melanoma cell lines, WM793B (human cells established from the vertical growth phase of a primary skin melanoma lesion) and 1205LU cell lines (lung metastasis of WM793B), were also assessed.

## Materials and methods

### Culture of human neonatal primary melanocytes (HNPMs)

T25 cell culture flasks (Sigma, Germany) and Petri dishes (55 mm in diameter; Sigma, Germany) were coated with human collagen type IV (0.67 µg/cm^2^; Sigma, Germany). The coated plastic materials were left inside a CO_2_ incubator for a minimum of 16 h. After this period, the excess collagen was removed, and the coated Petri dishes were left to dry inside a laminar flow chamber for 2 h. All coated consumables were kept at 5 °C if not used immediately. Before HNPMs thawing, the collagen-coated T25 flasks were washed with Dulbecco’s phosphate-buffered saline (D-PBS) (Invitrogen, USA). Thawing of HNPMs from the foreskin was performed according to the protocol provided by their supplier (Coriell Institute, USA). Medium 254 (Alfagene, USA) supplemented with human melanocyte growth supplement (HMGS; Alfagene, USA) at 37 °C was placed in the collagen-coated T25 flasks and left in the CO_2_ incubator for 16 h. After this period, the medium was replaced by 5 mL of fresh medium. When reaching 80% confluence, cells were washed with 3 mL of D-PBS and rinsed for a maximum of 5 min with 3 mL of versene (Invitrogen, USA). The previous solution was removed and 1 mL of a mixture of 0.05% trypsin (v/v) and 0.53 mM ethylenediaminetetraacetic acid (EDTA) (Gibco, USA) was added. The previous solution was neutralized with 5 mL of soybean trypsin inhibitor (Invitrogen, USA) and cells were centrifuged at 1200 rpm for 5 min. Cells (8 × 10^4^) were seeded in collagen-coated Petri dishes and kept inside the CO_2_ incubator at 37 °C overnight before mechanical properties were determined by AFM.

### Culture of human melanoma cell lines

The WM793B and 1205LU cells were seeded in coated Petri dishes (IBIDI, Germany). Cells were kindly provided by the Biophysics Institute (University of Bremen, Germany). Both cell types were cultured in DMEM (Merck, Germany), supplemented with 10% fetal bovine serum (FBS; Merck, Germany) and 1% penicillin/streptomycin (pen/strep) (Merck, Germany). Cell subculturing was performed after trypsinization with 0.05% (v/v) trypsin/0.53 mM EDTA solution (Merck, Germany), followed by washing steps with PBS without Ca^2+^ or Mg^2+^. Cells (8 × 10^4^) were then seeded in treated Petri dish and incubated for 24 h in the CO_2_ incubator at 37 °C before measurements.

### Assessment of cell mechanics using AFM (static and dynamic methodologies)

Assessment of mechanical properties by AFM was performed as described in Brás et al*.* [25]. Briefly, force maps of single force-distance curve maps (6 × 6 pixels), followed by force maps of frequency sweep force-distance curves maps (4 × 4 pixels), were obtained on a cell area 5 × 5 μm^2^, pointed at the nuclei of HNPMs and melanoma cell lines. The trigger point (maximum cantilever deflection) used was 3 nm. A scan rate of 2 Hz and velocity of 14.0 µm/s were used. In frequency sweep, the cantilever experienced in-phase and out-of-phase deflection signals, which were used to determine the complex shear modulus, creep compliance, and stress relaxation, as described by De Sousa et al*. *[27]. At the end of a conventional force-distance curve, a frequency sweep between 1 and 1000 Hz, with a dwell time of 8.7 s, was applied with a trigger point of 3 nm and an amplitude of 30 nm [27]. At least 40–70 cells were measured. The frequency sweep method allowed the determination of the power-law exponent of the storage modulus as a function of frequency [25]. AFM measurements were always performed after the 2nd passage of the cells. The parameters used in the Hertz fit as well as in the dwell window were selected according to Brás et al*.* [25]. A b-hydrodynamic factor of cantilever was calculated as described in Alcaraz et al. [28]. In short, the response of the cantilever was recorded at several distances from the sample (0, 200, 300, 600, 1100, 2100, 3600, and 5100 nm), while the z-height of the sample was modulated at several amplitudes (50, 100, 200, and 500 nm) in 254 or DMEM culture media, since their viscous properties (here translated by the loss modulus), as well as the viscous component of the cantilever, could be different. Following the procedure of Alcaraz et al*.*, the hydrodynamic correction factor b to a distance of 0 nm from the sample was extrapolated. This correction was used when fitting frequency data of cells was performed [28]. In the dwell window, the b-hydrodynamic factor introduced was 7.43 × 10^−7^. Before cell measurements, the calibration of the cantilever (model PFQNM-LC-A-CAL; Bruker, USA) was performed according Bras et al*.* [25]. Example of a conventional force-distance curve, both fitted with the Hertz model and a frequency sweep deflection as a function of time, using 1205LU cells, are presented in Fig. S1 (Supplementary Materials).

The loss and storage moduli were calculated for each frequency (see Fig. S2 in Supplementary Materials). However, since the loss and storage moduli follow a power-law behavior, it was considered sufficient to attribute the value of, for instance, the storage modulus at an arbitrary frequency and power-law exponent. We have selected 1 Hz, as this was the lowest frequency used.

We have fitted the frequency-dependent storage and loss modulus with the following equation, which is based on the hydrodynamic drag corrected version of the structural damping model as suggested by Alcaraz et al. [28]:

1 $${E}_{(\omega )}^{*}=\left({E}_{\left(\omega \right)}^{^{\prime}}+ i * {E}_{\left(\omega \right)}^{^{\prime\prime}} \right){\left(\frac{\omega }{{\omega }_{0}}\right)}^{\alpha } +i* {b}_{0}{\left(\frac{\omega }{{\omega }_{0}}\right)}^{1} +i* \mu {\left(\frac{\omega }{{\omega }_{0}}\right)}^{1}$$

where E* is the complex modulus, *E*′ and E″ are the storage and the loss moduli, respectively, *ω*_0_ is an arbitrary frequency constant, which was here set to 1 Hz, *b*_0_ is the hydrodynamic damping of the cantilever and *μ* is a Newtonian viscous term. Alternatively, Eq. 1 can be rewritten to use the absolute value of the modulus *E*^0^ and the loss tangent *η*:

2 $${E}_{\left(\omega \right)}^{*}={E}_{\left(\omega \right)}^{0}\left(1+ i\;tan(\eta ) \right){\left(\frac{\omega }{{\omega }_{0}}\right)}^{\alpha } +i* {b}_{0}{\left(\frac{\omega }{{\omega }_{0}}\right)}^{1} +i* \eta {\left(\frac{\omega }{{\omega }_{0}}\right)}^{1}$$

The apparent Young’s, storage and loss moduli, loss tangent, and the exponent of storage modulus of cells, were determined. The latter one was obtained in the frequency sweep, through the calculation of the power law of the storage modulus, to enlighten the dynamic behavior of cells. Previous works correlated the power-law exponent applied to the storage modulus to the “fluidity” of the cell cytosol [22–24].

The quantification of the viscoelastic properties from the frequency sweep of the curve corresponds to the slow response behavior of cells, in the frequency range 1–100 Hz. In the present work, only one power-law exponent was measured, obtained from the frequency sweep. The power-law exponent showed how much the storage modulus changed as a function of frequency (being frequency-dependent).

### Immunocytochemistry of melanoma cells

Human melanoma cells were seeded on coverslips at a cell density of 3 × 10^4^ cells/mL and incubated for 24 h at 37 °C and 5% CO_2_. After being washed with PBS, cells were fixed with 4% (v/v) paraformaldehyde (PFA; Electronic Sciences Microscopy, USA) in PBS for 20 min at room temperature (RT). Then, cells were washed 3 times with PBS for 5 min and maintained on the orbital shaker at 70 rpm. Next, cells were incubated with 0.25% (v/v) Triton X-100 (ITW, USA) in PBS for 5 min at RT. Human melanoma cell structures stained were nuclei, F-actin filaments, and p-FAK. For this purpose, samples were washed 3 × with PBS for 5 min and incubated with 1% (w/v) bovine serum albumin (BSA; VWR, USA) for 1 h at RT, involved in aluminum foil for protection from light. Subsequently, samples were incubated in 1% (w/v) BSA solution in PBS (1 ×) together with the primary antibodies anti-FAK (1:100, c-terminal rabbit; Invitrogen, Germany) at 4 °C during 3 h, under shaking at 70 rpm. The conjugated probe phalloidin/Alexa Fluor 488 (Molecular Probes-Invitrogen, USA) at 1:100 dilution for F-actin staining was added with the primary antibodies. Samples were then washed 3 × with PBS for 5 min and kept overnight at 4 °C for 18 h. The secondary antibody goat anti-rabbit Alexa Fluor 594 F(ab′)_2_ fragment was added for 3 h at RT (1:1000). Samples were then washed 3 × with PBS for 5 min under shaking of 70 rpm. The nuclei were counterstained with 4′,6-diamidino-2-phenylindole dihydrochloride (DAPI; H-1200, Vector, Germany) before being observed in a confocal scanning laser microscope (model SP3; Leica Microsystems, Germany). Cells were imaged with an HCX PL APO CS 40 × NA 1.3, using an oil objective (Leica Microsystems, Germany). Fluorescence data were visualized with ImageJ software (version 1.53C). Three immunohistochemistry experiments were performed for each cell type.

### Cell migration assay

Melanoma cell migration assays was carried out by measuring the surface area that cells occupy over time after creating a cell-free area through the scratch assay. Cells were seeded on Petri dishes and were kept in the CO_2_ incubator until confluency was reached. A 200-µL tip was then used to create a scratch with a width of approximately 2500 μm. Five replicates of each melanoma cell line were prepared, each one in 1 Petri dish. Cells were placed in an inverted microscope (Zeiss, Germany) and images were taken every 5 min, for 23 h to follow their migration. The images were analyzed using ImageJ software (1.53u version), using the wound healing tool script.

### Statistical analysis

The median and the 25th and 75th percentiles were used to visualize differences between cells. The Wilcoxon rank test was used to analyze the significance of differences. All statistical analyses were performed using the software package IGOR Pro 7 (WaveMetrics, USA). Statistically significant differences were indicated with (^***^); i.e., the difference between groups of distinct cells was statistically significant at *p* < 0.001.

## Results

### Analysis of the elasticity and viscoelastic properties

**Fig. 1:**
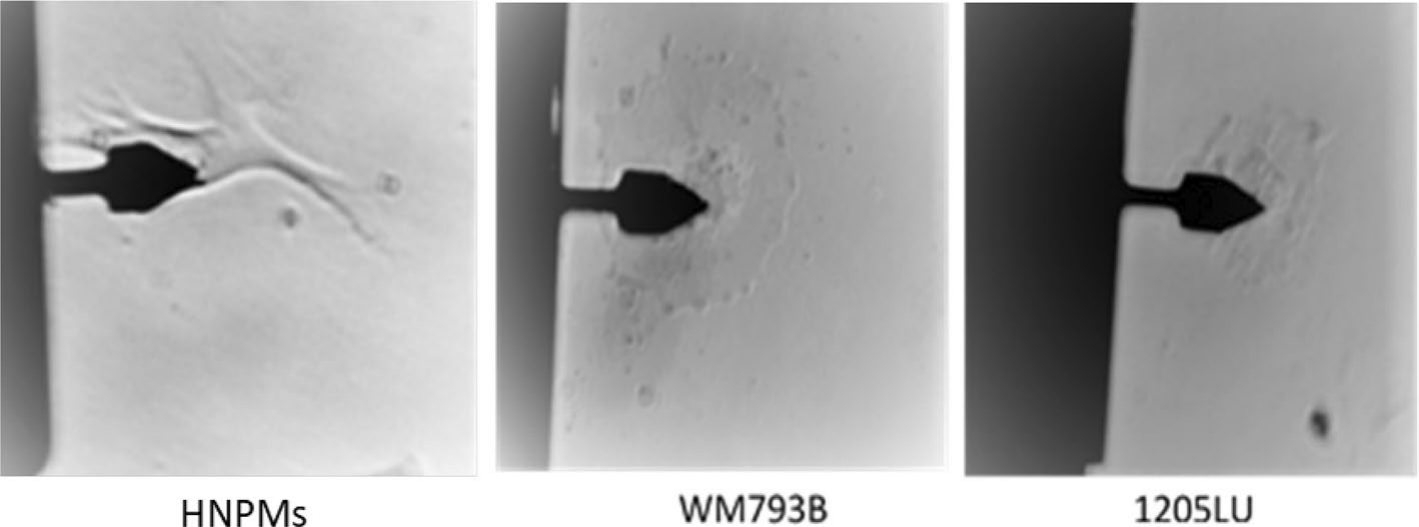
Representative examples of indentation using AFM cantilever PFQNM-LC-A-CAL to quantify the mechanical properties of HNPMs, WM793B, and 1205LU cells. The tip pointed at the cell nucleus

Aiming to analyze the mechanical properties of HNPMs and melanoma cancer cells, the tip was placed above the nucleus of each cell type (Fig. 1), and a force map was obtained from conventional force-distance curves acquisition, followed by a frequency sweep map with a dwell time of 8.7 s.

**Fig. 2:**
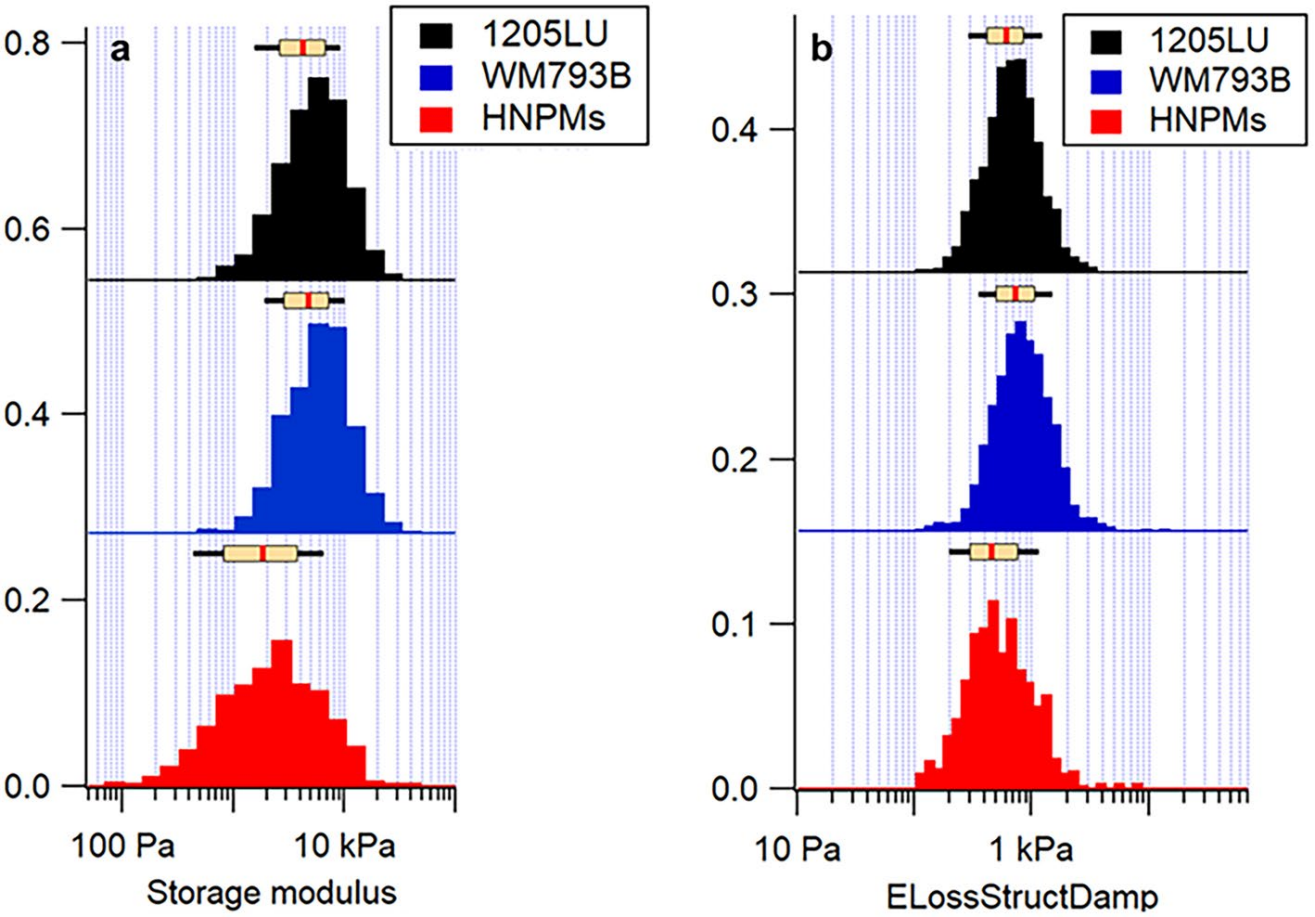
Histograms of mechanical properties quantification of HNPMs (red), WM793B (blue), and 1205LU (black) cells: **a** Storage modulus; and **b** loss structure damping modulus. The box plot above the histograms present the median (red line) and the 25th and 75th percentiles (extent of rectangle in cream color), and the 10th and 90th percentiles (black lines extending beyond the box)

**Fig. 3:**
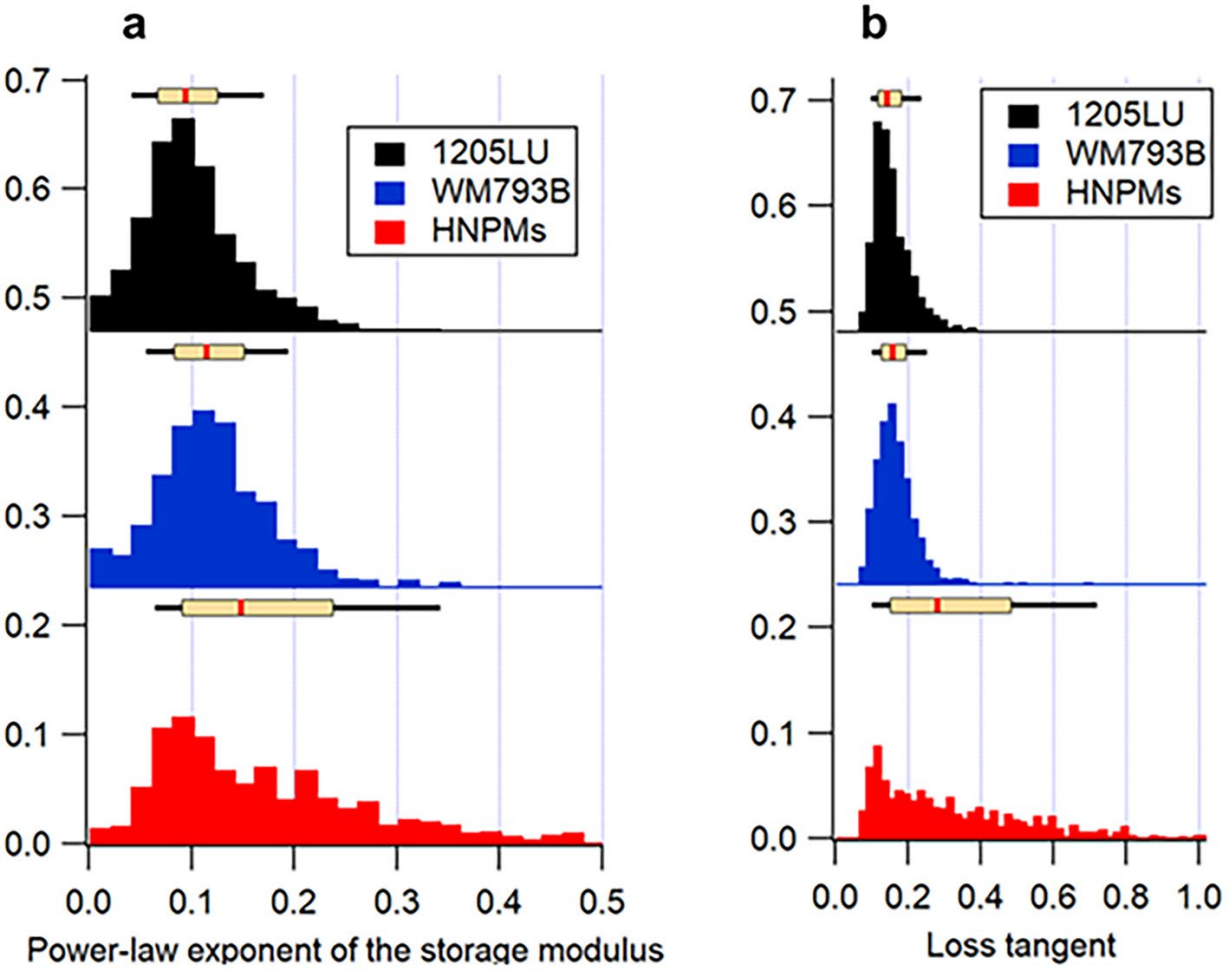
Histograms of mechanical properties quantification of HNPMs (red), WM793B (blue), and 1205LU (black) cells: **a** Power-law exponent of the storage modulus; and **b** loss tangent. The box plot above the histograms present the median (red line) and the 25th and 75th percentiles (extent of rectangle in cream color), and the 10th and 90th percentiles (black lines extending beyond the box)

Then, histograms were obtained (Figs. 2, 3 and S3 (in Supplementary Materials)), and statistical data treatment was performed. Data is presented as a boxplot with median and min-to-max whiskers for all cells (40 to 70 cells measured).

The quantification of apparent Young’s modulus and parameters obtained with frequency sweep for HNPMs, WM973B, and 1205LU cells are presented in Table S1 (in Supplementary Materials).

As can be seen in Table 1, the apparent Young’s and the storage moduli were significantly different when comparing HNPMs cells with WM793B and 1205LU cells. However, there were no significant differences between WM793B and 1205LU cells.

**Table 1:** Statistical data treatment of HNPMs and melanoma cell lines

**Mechanical properties**	**HNPMs/WM793B**	**HNPMs/1205LU**	**WM793B/1205LU**
Apparent Young’s modulus (kPa)	7.21 × 10^−9***^	2.71 × 10^−7***^	0.11
Storage modulus (kPa)	6.20 × 10^−12***^	1.20 × 10^−11***^	0.14
Loss modulus (kPa)	6.7 × 10^−7***^	0.007	5.00 × 10^−4***^
Power-law exponent of storage modulus (^***^)	1.28 × 10^−5^	2.22 × 10^−4^	3.61 × 10^−10^
Loss tangent	1.61 × 10^−6***^	3.61 × 10^−10***^	0.003

The Wilcoxon rank test was used

^***^Corresponds to statistically significant differences between HNPMs and melanoma cell lines (*p *< 0.001)

In terms of loss modulus, there was no significant difference between the HNPMs and 1205LU cells, despite the former ones being healthy cells and the latter ones being a tumor metastatic cell line. This could be explained by the fact that HNPMs are neonatal cells, and thus naturally softer. This means that the viscous properties of these cells are similar; i.e., when cells were probed by the AFM tip, the movements of the cytosol and their components behave in the same way. Regarding the power-law exponent of the storage modulus, values obtained presented a decreasing order (0.13, 0.11, and 0.08) regarding HNPMs, WM793B, and 1205LU cells, respectively. Table 1 shows statistical differences in the power-law exponent of the three cell types which may also be related to the loss tangent. The loss tangent of HNPMs was significantly different from that of WM793B and 1205LU cells. However, the loss tangent did not show significant differences between WM793B and 1205LU cells. This corroborates the inexistence of significant differences in the storage modulus between WM793B and 1205LU cells since the power law was calculated through the storage modulus. HNPMs are statistically different from the WM793B and 1205LU cells regarding all mechanical parameters, except in viscous properties, which were similar for HNPMs and 1205LU cells.

Table S1 shows data in more detail, namely that the apparent Young’s modulus of HNPMs was lower (2.94 kPa), when compared with the melanoma cells (4.58 kPa for WM793B and 3.89 kPa for 1205LU). The storage (1.94 kPa, 4.69 kPa, and 4.27 kPa, respectively) and the loss (0.52 kPa, 0.73 kPa, and 0.61 kPa, respectively) moduli showed the same trend as the apparent Young’s modulus. For HNPMs, the loss modulus was lower by a factor of 3.7, when compared with the storage modulus. However, for WM793B and for 1205LU, the loss modulus was lower by factors of 6.4 and 7, respectively, when compared with the storage modulus.

### Cytoskeleton protein expression and localization and cell migration

In this study, the cytoskeleton expression and localization and cell migration of HNPMs were not performed, since the focus of the work was the microrheology AFM between melanoma cell lines.

**Fig. 4:**
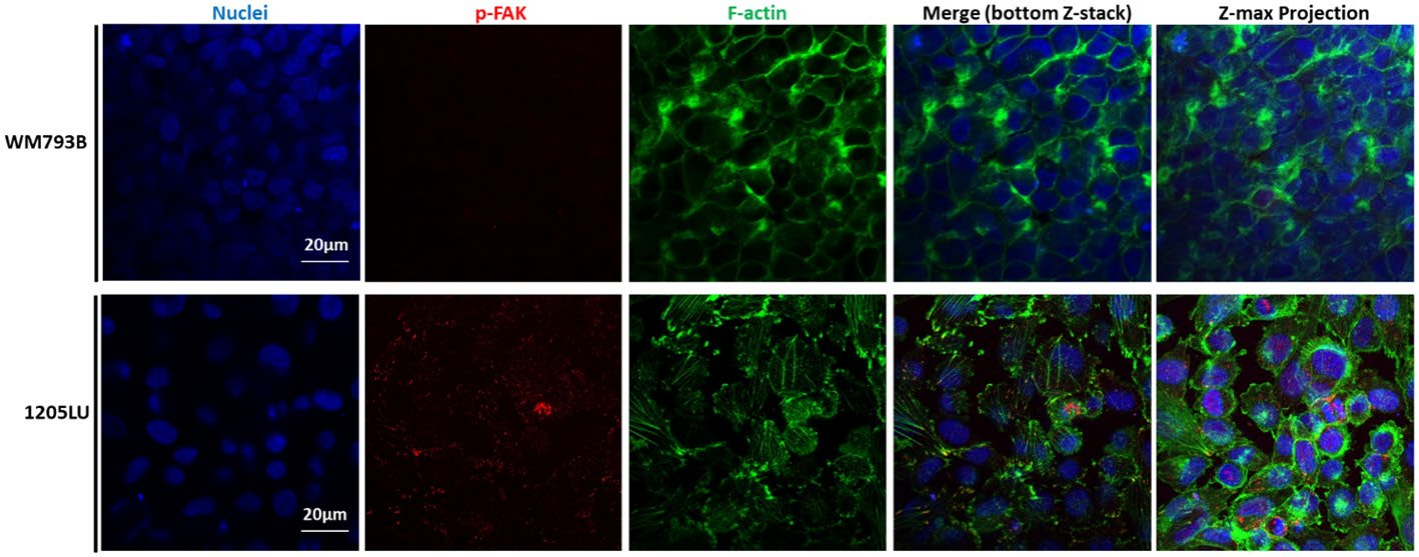
F-actin distribution in melanoma cell lines (WM793B and 1205LU). Staining of cells for DAPI to identify the cell nuclei (blue), p-FAK (red), and phalloidin from F-actin filaments (green). The overlap of the three channels is presented in the bottom Z-stack and the Z-maximum projection of the imaged area. Scale bars: 20 µm

The assessment of the mechanical properties by AFM showed interesting differences between melanoma cell lines, despite being both cancer cells. To better understand the relation between the mechanical properties and the morphodynamic properties of the melanoma cell lines, WM793B and 1205LU, we used immunofluorescence to evaluate cytoskeletal organization with F-actin labelling and focal adhesion localization with p-FAK staining and a migration assay to assess the migratory cell behavior. As can be observed in Fig. 4, F-actin was clearly present, forming a cortical ring in WM793B cells, while in 1205LU cells, F-actin was organized in stress fibers throughout the cytoplasm and in lamellipodia, not showing a cortical distribution. Using p-FAK staining as a proxy of activated focal adhesions (one of the important structures for cell motility), it could be observed in the metastasis-derived 1205LU cells that p-FAK was localized at the tip of several F-actin filaments (stress fibers) and lamellipodia, typical of the focal adhesion complexes (Fig. 4, bottom Z-stack). On the contrary, in WM793B cells, p-FAK could not be detected. Given this different organization of the F-actin in the melanoma cell lines, and taking into account that the presence of stress fibers and activated focal adhesions are both important for cell contractility to provide the force for cell migration, both cell lines were assayed for cell migration.

**Fig. 5:**
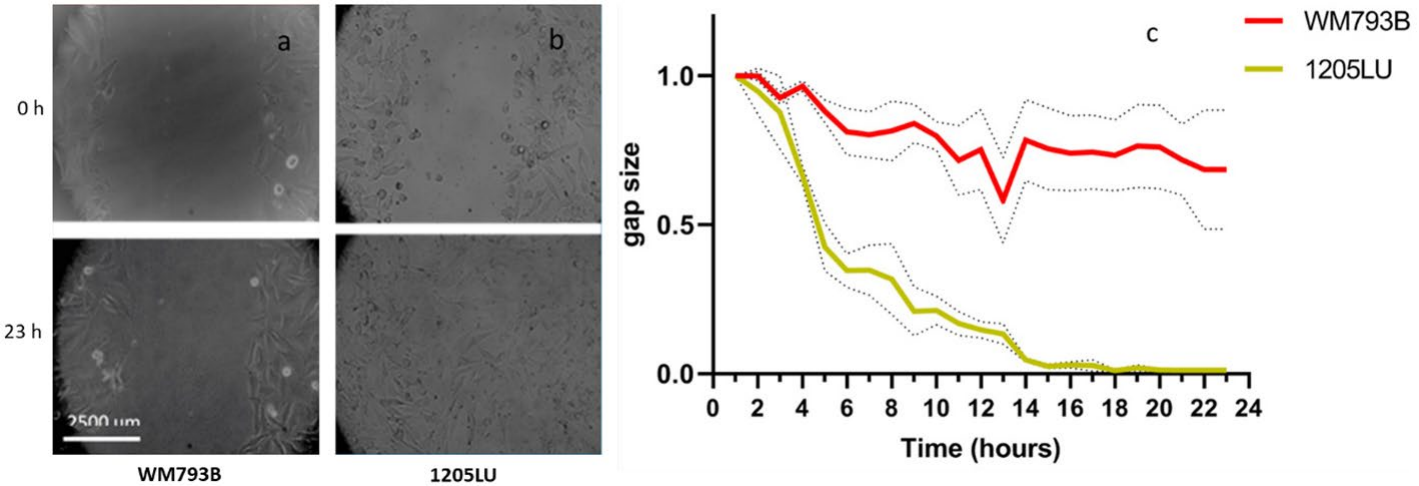
Cell migration assay of WM793B and 1205LU cell lines between 0 and 23 h. **a** WM793B cell line. **b** 1205LU cell line. **c** Gap size (i.e. state of closure) during the 23 h of the assay, for both cell lines

As shown in Fig. 5, at the end of the assay, the 1205LU metastatic cell line completely closed the gap, while WM793B only closed 30% of the gap, thus indicating that 1205LU migrated more than WM793B cells. Table 2 summarizes all the results obtained, namely the mechanical measurements, cytoskeleton structure, and p-FAK, as well as cell migration assays.

**Table 2:** Summary of the results related to mechanical properties, cytoskeleton organization structure and p-FAK

**Analyzed parameters**	**HNPMs**	**WM793B**	**1205LU**
Apparent Young’s modulus (kPa)	2.94	4.58	3.89
Storage modulus (kPa)	1.94	4.69	4.27
Loss modulus (kPa)	0.52	0.73	0.61
Power-law exponent of the storage modulus	0.13	0.11	0.08
Loss tangent	0.25	0.16	0.12
F-actin	NP	Abundant cortical actin	Distributed through the whole cell
p-FAK	NP	(-)	(++)
Wound healing (23 h)	NP	Did not close	Closed

(-) absence, (+) low, (++) intermediate, *NP* not performed

## Discussion

In published works related to the quantification of mechanical properties of tumor skin cells, adult primary cells are usually selected as control cells. An example can be found in the work performed by Bobrowska et al*.*, in which it can be seen that the Young’s modulus of adult primary cells were stiffer compared with the tumor skin cells [18]. In our work, HNPMs were chosen as the control cells. The goals of this work were as follows: (i) to compare the mechanical properties of human neonatal primary melanocytes (HNPMs) with that of two melanoma cell lines (WM793B and 1205LU cells), using a new AFM dynamic approach, which allows the quantification of not only the viscoelastic properties of cells but also the power-law exponent of the storage modulus (provides an information on how the storage modulus is frequency-dependent); and (ii) by using cell biophysics, mainly in microrheology AFM between melanoma cell lines, to characterize cancer cells in terms nanomechanics.

In terms of indentation depth, the force-distance curves were performed with the maximum deflection of 3 nm, which results in a maximum force of 0.3 nN. Depending on the stiffness (and the tip geometry), this will result in an indentation of around 300 nm. However, the deformation depth, i.e., the volume that is actually deformed (and hence contributes to the mechanical response), is much larger. It is conceivable that this volume has a depth of 5–10 times the indentation, since this is also the range where a bottom effect is felt. Thus, the mechanical response will not only come from the actin cortex, but also deeper areas of the cell.

The vast majority of the published literature aiming to measure the mechanical properties of cells assumes that cells are purely elastic, semi-infinite, homogeneous, and isotropic models, which is far from reality [18–20]. In fact, cells are dynamic entities that can be characterized by elasticity, viscous properties, glassy state, and power-law exponent [24, 26]. As it has been shown during this study, dynamic AFM frequency sweep confirmed the existence of creep and stress relaxation of cells (see Fig. S1, black arrow, in Supplementary Materials), which corroborated that cells are complex entities by not behaving elastically. The characterization of mechanical properties was performed using the determination of the apparent Young’s modulus and viscoelasticity, through the acquisition of conventional force-distance curves and frequency sweep, respectively. The frequency sweep allowed the determination of the storage and loss moduli, the loss tangent, and the power-law exponent applied to the storage modulus. The presence of a glassy phase indicates if the cells are more solid-like (if the value is close to 0) or liquid-like (if the value is closer to 1). In this work, the higher was power-law exponent of the cells, the higher was their motility, thus increasing their potential to form metastasis [29]. The quantification of the viscoelastic properties is very important regarding cell proliferation, motility, and regulation of disease state [30]. According to de Sousa et al., there are studies mentioning that cells present two power-law shear moduli, namely high and low exponents corresponding to the fast and slow cell response, respectively [26]. In this work, a power law applied to the storage modulus of the frequency sweep was calculated, corresponding to the slow response of the cell [26]. The slow response is assigned to the glassy-like regimen of the cytoskeleton dynamics in cell mechanics [26].

Considering the obtained data in Table 2, the results in terms of mechanical properties can be summarized as follows:

- Apparent Young’s modulus: WM793B > 1205LU > HNPMs
- Storage modulus: WM793B > 1205LU > HNPMs
- Loss modulus: WM793B > 1205LU > HNPMs
- Power-law exponent of the storage modulus: 1205LU < WM793B < HNPMs
- Loss tangent: 1205LU < WM793B < HNPMs

Bobrowska et al. compared the apparent Young’s modulus of melanocytes from adult skin with several melanoma cells, including WM793B and 1205LU [31]. The apparent Young’s modulus of adult melanocytes follows the opposite trend when comparing the values regarding the HNPMs of our work. This can be explained due to the difference in sources of primary melanocytes. While the ones used here were from foreskin of a 1-year-old child, those from Bobrowska and co-workers were from adult skin [18].

Cells with low Young’s modulus are claimed to exhibit great potential to invade tissues, supporting the relevance of studying biophysical properties, such as cell elasticity of the different malignant cells [32]. Weder et al. demonstrated by using AFM that the transformation of the radial growth phase to the vertical growth phase contributed to melanoma progression that was associated with a decrease in cell stiffness, which promoted cell deformation and invasion into the surrounding stroma, as well as increased intravasation [33]. The storage modulus was correlated with the amount, distribution, and cross-linking of F-actin filaments since these filaments were claimed to be, to a large extent, responsible for the resistance of cells to deformation/indentation; i.e*.*, it was claimed to correspond to a measure of cell elasticity [34]. As previously discussed, melanoma cells presented higher storage modulus (4.69 kPa for WM793B and 4.27 kPa for 1205LU, compared with 1.94 kPa for HNPMs). The difference between these values could be explained due to use of colloidal probes, which have a large hydrodynamic drag, making it more difficult to measure the viscous properties of soft materials. In addition, the experimental scheme, based on taking regular force curves and analyzing the response of the sample during loading, was also very sensitive due to misjudging the exact time of contact.

Regarding the exponent of the storage modulus, HNPMs showed the highest value (0.13) when compared with WM793B (0.11) and 1205LU (0.08) cells. According to Zhang et al., this parameter can be correlated with the cell cytosol “fluidity” [35]. These results agree with the fact that WM793B and 1205LU were found to have a lower power-law exponent, corresponding to the fact that these cells were stiffer (storage modulus values of 4.69 and 4.27 kPa, respectively) when compared with HNPMs (1.94 kPa). Sanchez et al. confirmed that the storage modulus varied inversely with the power-law exponent [36]. The loss tangent, which is based on the ratio between the loss and the storage moduli (corresponding to the glassy phase), is correlated with the solid-like or liquid-like state of the cell [27].

According to Table S1, the apparent Young’s modulus of HNPMs and melanoma cells was significantly different, except in the viscous properties between HNPMs and 1205LU, where similar values were obtained. There were no significant differences between WM793B and 1205LU cell lines, either in the apparent Young’s modulus or storage modulus, which can likely be explained by the fact that these cells are cancer cells and potentially have a similar origin, since 1205LU are lung metastasis from WM793B cell line. Importantly, significant differences between the power-law exponent of the storage modulus and the loss tangent were observed, except between WM793B and 1205LU cell lines. These cells presented values of loss tangent of 0.16 and 0.12, respectively. They are more liquid-like, which is in accordance with their elasticity, translated by their storage modulus. This fact could mean that both melanoma cells are more liquid-like (when compared with HNPMs), which may explain the same trend followed in terms of storage modulus (4.69 kPa and 4.27 kPa, respectively). The similarity of power-law and loss tangent could be explained by an eventually similar state of the cytoskeleton and distribution of the organelles inside these cells, and the way the cytosol diffusion occurs through the various components. Wang et al. concluded that similar tumor cells present significant variations of cytoplasmic viscous properties within the same cell type, using micropipette aspiration [37]. Kwapiszewska et al. highlighted that the viscous properties of the cytoplasm are not constant since they depend on the size of the components inside the cells, mentioning that their increase is correlated with the increase in organelle size [38]. The various organelles constitute an obstacle to probe indentation. The cytosol must flow through (or around) the organelles, which is related to cytosol fluidity.

Since interesting differences were found in AFM mechanical properties regarding WM793B and 1205LU cell lines, qualitative immunocytochemistry and cell migration assays of melanoma cells were performed in an attempt to correlate them. The mechanical properties were thus correlated with the cytoskeleton structure, focal adhesion, and migratory behavior. Regarding the cytoskeleton structures, WM793B cells presented cortical F-actin filaments (Fig. 4), while 1205LU cells presented F-actin distributed along the cell body, organized in stress fibers throughout the cytoplasm and lamellipodia structures, without cortical distribution. Regarding p-FAK, it was absent in WM793B, while it was present in 1205LU cells (Fig. 4). Cells adhere to ECM or substrates using molecular adhesions, such as focal adhesions (FAs), FA complexes, or podosomes [39]. The presence of p-FAK in 1205LU cells (Fig. 4), associated with their gap closure ability after 23 h, correlates well with their migratory behavior (Fig. 5). Kahana et al. pinpointed that FAK might have a possible role in melanoma progression [40]. Interestingly, the presence of p-FAK correlated with the highest migratory behavior of 1205LU (where the gap was 100% closed after 23 h) compared with WM793B cells (where the gap was only ca. 30% closed after the same time period). This higher capability to migrate is not in accordance with the observed power-law exponent. Unexpectedly, 1205LU cells presented lower power-law exponent than WM793B cells. This fact indicates that other mechanisms probably also play a role in the mechanical properties of melanoma cells. In terms of WM793B cell line, they lacked p-FAK and it was observed that they only closed 30% of the gap after 23 h, which is in agreement with the result obtained (37%) for the same cells found by Wasinger et al. [41].

In terms of power-law exponent, HNPMs showed a higher value, being more liquid-like than the other cells studied. WM793B and 1205LU cells showed the same trend in terms of apparent Young’s, storage and loss moduli but not regarding the power-law exponent of the storage modulus (Tables 2 and S1).

## Conclusions

HPNMs cells presented statistically significant differences in mechanical properties when compared with WM793B and 1205LU in all parameters, except in terms of viscous properties that are comparable to 1205LU. WM793B and 1205LU presented similar in Young’s storage moduli, but differed in viscous properties and power-law exponent. This could be understandable with the eventual differences in terms of organelle size and how they move and rearrange themselves inside the cell cytosol, and how the latter flows through, responding to the tip stress during the indentation.

Interestingly, although the power-law exponent value of 1205LU was lower than that of WM793B cells, they presented a higher migratory behavior, which can be related to the presence of p-FAK and high levels of stress fibers. Eventually, the internal forces generated by this cytoskeleton rearrangement of 1205LU cells compensated the low power-law exponent. Nevertheless, the cells originating from the primary tumor are more elastic and viscous and thus less deformable than the corresponding metastasis.

The innovative dynamic AFM methodology applied to HNPMs and melanoma cells allowed the quantification of elasticity, viscous properties, glassy phase, and power-law properties (the latter can be translated into “fluidity” of the cell cytosol, according to some studies), which may have an impact in tumor cell properties such as migration and metastasis. The study of dynamic mechanical properties of melanoma cells is important since they can be used as a biomarker of the different stages of the disease, taking into account that this cancer type is one of the most aggressive. This work showed that the viscous properties seem to contribute less to mechanical properties when compared to elasticity and the power-law exponent. Those last two parameters may be used as a biomarker of pathological cell state and potential to form metastasis. This work also showed that microrheology AFM methodology can be applied to other cancer cell types was also observed by Brás et al*.* [25].

### Supplementary Information

Below is the link to the electronic supplementary material.

Supplementary file1 (DOCX 618 KB)
