# Supplementary material for: Microrheological comparison of melanoma cells by atomic force microscopy
Source: J Biol Phys. 2024 Jan 19;50(1):55–69. doi: 10.1007/s10867-023-09648-w (PMC10864228; doi:10.1007/s10867-023-09648-w)
Supplement: Supplementary file 1 — Supplementary file1 (DOCX 618 KB) [file 10867_2023_9648_MOESM1_ESM.docx]

**Supplementary Materials**


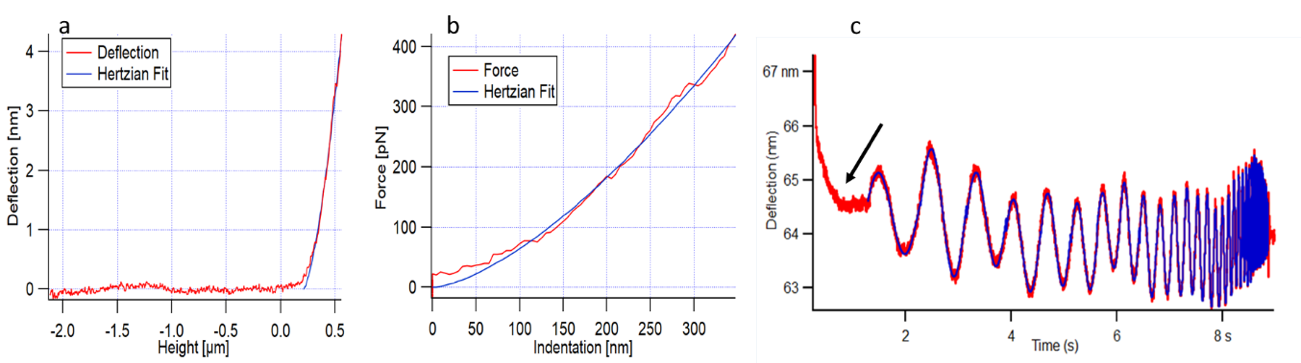


**Figure S1.** Examples of a conventional force-distance curves on a 1205LU cell, both fitted with Hertz’s model and a frequency sweep deflection as a function of time**:** a) Deflection *vs*. height approach curve; b) force *vs*. indentation curve; c) representative curve of the deflection signal *vs.* time during dwell (tip contact for 8.7 s), in frequency sweep modulation. The first region of red curve (black arrow) corresponds to cells’ stress relaxation and creep of the cell when the tip touches the surface during tip approach. The analysis of the curve started with the curve fitting (blue color).


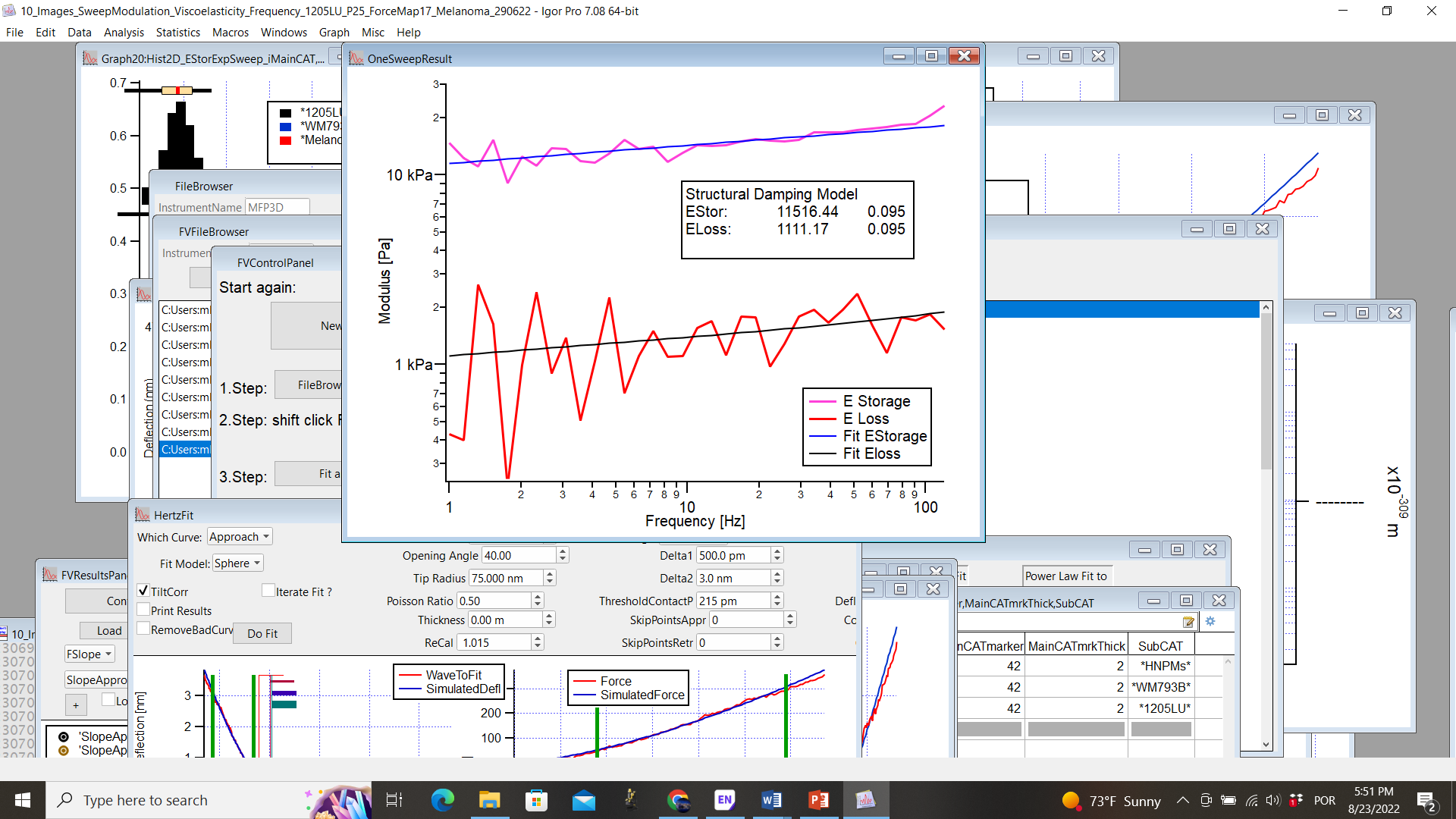


**Figure S2.** Representative example of loss and storage moduli *vs*. frequency curve, obtained by frequency sweep in a 1205LU cell. The structural damping model was fitted to the data, which uses the same power-law exponent for storage and loss modulus plus a Newtonian viscous term. In this data set, we have obtained from the fit a storage and loss moduli values for the lowest frequency (1 Hz) around 11.5 kPa and 1.1 kPa, respectively, and a power-law exponent of 0.095. Since the loss and storage moduli follow a power-law behavior, it is sufficient to give the value of e.g. of the storage modulus at an arbitrary frequency and power-law exponent. We have chosen 1 Hz, as this is the lowest frequency used. Despite applying a correction for the hydrodynamic drag of the cantilever, the apparent loss modulus shows additional viscous damping at higher frequencies, which is taken care of by the Newtonian viscous term of the structural damping model (see main text eq. 1).


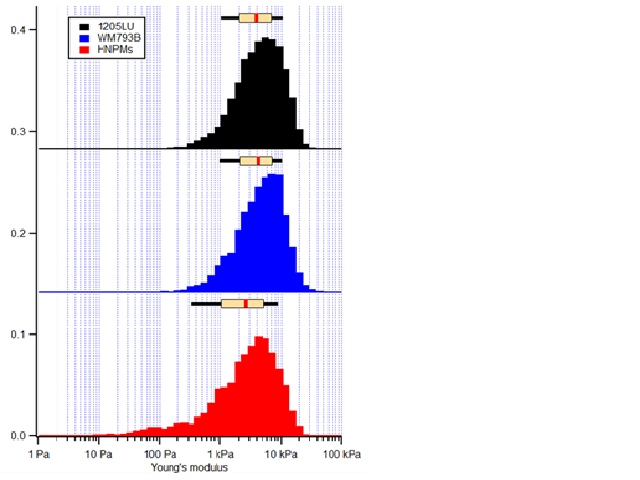


Figure S3. Histogram of Young’s modulus quantification of HNPMs (red), WM793B (blue), and 1205LU (black) cell.

**Table S1.** Values of median and percentiles of Young’s, storage, loss moduli, and power-law exponent applied to the storage modulus and loss tangent of HNPMs, WM973B, and 1205LU cells, quantified by AFM.

| **Cell type** | **HNPMs** | | | **WM793B** | | | **1205LU** | | |
| --- | --- | --- | --- | --- | --- | --- | --- | --- | --- |
| **Mechanical properties** | **25th** | **Median** | **75th** | **25th** | **Median** | **75th** | **25th** | **Median** | **75th** |
| Apparent Young’s modulus (kPa) | 1.68 | **2.94** | 4.34 | 2.79 | **4.58** | 6.28 | 2.47 | **3.89** | 6.38 |
| Storage modulus (kPa) | 1.32 | **1.94** | 2.81 | 3.55 | **4.69** | 6.39 | 2.92 | **4.27** | 6.09 |
| Loss modulus(kPa) | 0.32 | **0.52** | 0.64 | 0.61 | **0.73** | 0.91 | 0.47 | **0.61** | 0.75 |
| Power-law exponent of storage modulus | 0.11 | **0.13** | 0.19 | 0.08 | **0.11** | 0.12 | 0.07 | **0.08** | 0.10 |
| Loss tangent | 0.18 | **0.25** | 0.44 | 0.14 | **0.16** | 0.18 | 0.12 | **0.13** | 0.17 |
